# Current Knowledge of Germline Genetic Risk Factors for the Development of Non-Medullary Thyroid Cancer

**DOI:** 10.3390/genes10070482

**Published:** 2019-06-26

**Authors:** Kinga Hińcza, Artur Kowalik, Aldona Kowalska

**Affiliations:** 1 Department Molecular Diagnostics, Holycross Centre, 25-734 Kielce, Poland; 2 The Faculty of Health Sciences of the Jan Kochanowski University, 25-317 Kielce, Poland; 3 Endocrinology Clinic of Holycross Cancer Centre, 25-734 Kielce, Poland

**Keywords:** thyroid cancer, genetic abnormalities, genetic markers, molecular testing, oncogenic mutations

## Abstract

The thyroid is the most common site of endocrine cancer. One type of thyroid cancer, non-medullary thyroid cancer (NMTC), develops from follicular cells and represents approximately 90% of all thyroid cancers. Approximately 5%–15% of NMTC cases are thought to be of familial origin (FNMTC), which is defined as the occurrence of the disease in three or more first-degree relatives of the patient. It is often divided into two groups: Syndrome-associated and non-syndromic. The associated syndromes include Cowden syndrome, familial adenomatous polyposis, Gardner syndrome, Carney complex and Werner syndrome. The hereditary factors contributing to the unfavorable course of FNMTC remain poorly understood; therefore, considerable effort is being expended to identify contributing loci. Research carried out to date identifies fourteen genes (*DICER1*, *FOXE1*, *PTCSC2*, *MYH9*, *SRGAP1*, *HABP2*, *BRCA1*, *CHEK2*, *ATM*, *RASAL1*, *SRRM2*, *XRCC1*, *TITF-1/NKX2.1*, *PTCSC3*) associated with vulnerability to FNMTC that are not related to hereditary syndromes. In this review, we summarize FNMTC studies to date, and provide information on genes involved in the development of non-syndromic familial non-medullary thyroid cancers, and the significance of mutations in these genes as risk factors. Moreover, we discuss whether the genetic polymorphism rs966423 in *DIRC3* has any potential as a prognostic factor of papillary thyroid cancer.

## 1. Introduction

**Figure 1:**
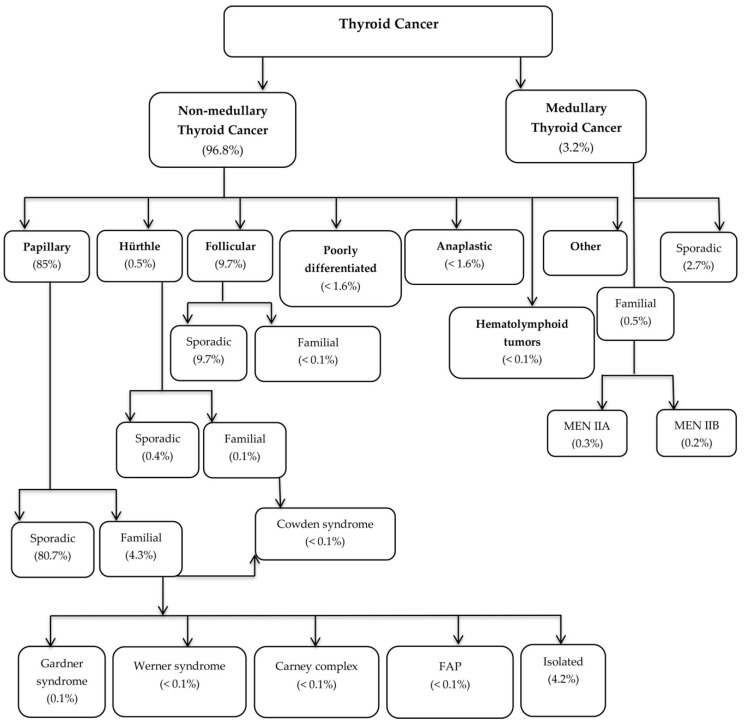
Thyroid Cancer classification (modified from Vriens et al. 2009).

Thyroid cancer is the most common malignant endocrine tumor, accounting for 1%–2% of all malignancies. In 2018, the World Health Organization (Global Cancer Statistics 2018: GLOBOCAN) reported 567,233 new cases of thyroid cancer, of which 41,071 resulted in death [1]. Thyroid cancer types are classified according to their histological characteristics (Figure 1) [2].

Malignant tumors of the thyroid gland can be derived from all types of cell present in this tissue. The vast majority are of follicular cell, parafollicular cell (C cell), and lymphoid cell origin, with tumors derived from other cell types occurring very rarely. Medullary thyroid carcinoma (MTC) develops from neuroendocrine C cells, and can occur sporadically or as a familial condition. The familial form of MTC accounts for 20%–25% of cases, and is usually a component of multiple endocrine neoplasia (MEN) IIA or IIB [3]. Differentiated thyroid cancer (DTC) of follicular cell origin is also known as non-medullary thyroid cancer (NMTC) and represents approximately 90% of all thyroid cancers, and approximately 5%–15% of NMTC cases are thought to be of familial origin (FNMTC) [4]. 

FNMTC has an autosomal dominant pattern of inheritance, and the disease can occur as a result of either heterozygous or homozygous mutations. Moreover, polygenic inheritance is also plausible, particularly in those cases where only two family members are affected. Given the fairly frequent occurrence of NMTC, a definitive diagnosis of FNMTC must be made with caution, particularly in families where only two members are affected. FNMTC cases are characterized by high genetic heterogeneity (molecular heterogeneity), making it difficult to identify key molecular changes [5]. To date, genetic alterations with key roles in the development of each FNMTC subtype have yet to be fully characterized, representing a significant problem for screening and estimating the prognosis for individuals with the disease. Moses et al. (2011) report no significant differences in the type or number of somatic mutations (in *BRAF*, *NRAS*, *KRAS*, *RET*, and *NTRK1*) between cases with sporadic and familial NMTC [5]. 

The clinical characteristics of FNMTC are controversial. Some, but not all, authors have reported an earlier age of onset, higher incidence of multifocality and lymph node metastasis, and a more aggressive outcome with more frequent relapses, compared with sporadic disease [5,6]. Similar findings are reported by El Lakis et al. (2019) in FNMTC families with three or more affected members, suggesting a more aggressive disease with a greater rate of lymph node metastasis and an earlier age of onset, relative to the sporadic form. 

Further, based on their results, these authors suggest that surgical treatment should be more aggressive for patients with FNMTC [7]; however, a retrospective study by Pitoia et al. (2011) did not detect statistically significant differences in age at diagnosis, sex, tumor size, tumor stage or distant metastasis between patients with familial and sporadic NMTC. The only differences in the baseline characteristics observed between the two groups were in bilateral malignancy (38% vs. 24%, respectively; *p =* 0.03), lymph node metastasis (56.2% vs. 39%, respectively; *p =* 0.01), and multicentricity (43% vs. 28%, respectively; *p =* 0.03) [6]; however, Moses et al. (2011) suggest that patients with FNMTC present at a significantly younger age (an average of 5 years younger) than those with sporadic thyroid cancer, although no other statistically significant differences are detected. It is possible that the earlier diagnosis in individuals with FNMTC can be attributed to greater awareness and better access to preventive and screening tests [5]. Given the small differences in the clinical characteristics of patients with familial and sporadic NMTC, the therapeutic strategy for both diseases is the same [8,9]. FNMTC is defined based on the occurrence of disease in three or more first-degree relatives of the patient, and is often divided into two groups: Syndrome-associated and non-syndromic [3,6]. 

## 2. Syndromic FNMTC

Inherited syndromes associated with an increased risk of FNMTC include the following: Familial adenomatous polyposis (FAP), Cowden syndrome, Werner syndrome, Carney complex, and papillary renal neoplasia (Table 1). Mutations in several genes are identified as responsible for these hereditary syndromes. Alterations in the *APC* gene are associated with FAP, and changes in *PTEN*, *SDHB-D*, *PIK3CA*, *AKT1*, *KLLN*, and *SEC23B* are responsible for Cowden syndrome. Mutations in *PRKAR1α* cause Carney complex, while disturbances of the *WRN* gene signaling pathway are associated with development of Werner syndrome. Patients with the above-mentioned hereditary syndromes exhibit increased incidence of both thyroid cancer and other types of malignancy [10,11,12,13,14,15,16,17,18,19,20,21,22,23,24]. 

### 2.1. Familial Adenomatous Polyposis 

FAP is an autosomal dominant disease caused by inactivating mutations in the *APC* tumor suppressor gene on chromosome 5q21. In addition to numerous intestinal polyps, colon cancer, and other neoplasms, some patients with FAP are at increased risk of developing thyroid cancer. Of patients with FAP, 1%–12% develop thyroid carcinoma. Further, thyroid carcinoma associated with FAP is very often multifocal, and is more common among women and people under 30 years of age [24,25]. 

### 2.2. Cowden Syndrome (PTEN Hamartoma Syndrome) 

Cowden syndrome is an autosomal dominant disorder characterized by hamartomatous changes and epithelial tumors of the breast, thyroid, kidney, colon and endometrium. *PTEN* gene mutations on chromosome 10q22–23 can cause this disease; however, in some cases, the cause is unknown, although these are also probably associated with a reduced PTEN protein suppressor activity. At least two thirds of patients with this syndrome are affected by thyroid disease, often before the age of 20 years. In addition, approximately 10% of patients with Cowden syndrome will develop thyroid cancer in their lifetime, usually papillary thyroid cancer (PTC) or follicular thyroid carcinoma (FTC) histological types. In addition, a comparison of the numbers of thyroid nodules show that patients with thyroid cancer from the general population have significantly fewer than those with Cowden syndrome [24,25].

### 2.3. Werner Syndrome

Werner syndrome is an autosomal recessive disorder caused by mutations in the *WRN* gene on chromosome 8p11.1–21.1. This syndrome is associated with premature aging, short stature, and several neoplasms, including meningiomas, soft tissue sarcomas, and thyroid carcinoma. 

Werner syndrome is particularly common in Japan, and Japanese patients with this condition are more likely to develop thyroid cancer than Caucasians with the syndrome. The histologic types of thyroid cancer detected in patients with Werner syndrome include FTC, PTC, and occasionally anaplastic thyroid cancer [24]. Approximately 18% of Japanese patients with Werner syndrome develop thyroid cancer [24,25]. 

### 2.4. Carney Complex

Carney complex is most commonly due to a mutation of the *PRKAR1A* gene on chromosome 17q22–24; however, some cases are linked with an unknown gene at 2p16. Carney complex is an autosomal dominant disease, in which patients have spotty skin pigmentation and an increased risk of cardiac myxomas, and develop a variety of tumors involving endocrine organs; almost 60% of patients with this condition develop tumors of the thyroid gland. Histologically, most nodules are benign adenomas; however, approximately 3% of patients will develop thyroid carcinoma, including PTC and FTC [24,25].

### 2.5. Papillary Renal Neoplasia (PRN) and Other Rare Syndromes Associated with NMTC

PRN is associated with the occurrence of malignant renal tumors. The genetic cause of PRN is not yet known; however, an unidentified gene at the chromosomal locus 1q21 is likely responsible for this autosomal dominant disease associated with papillary renal carcinoma and thyroid cancer. A PTC phenotype associated with PRN is extremely rare, and is only described in one family to date [24]. 

Several other hereditary conditions, including McCune-Albright, Peutz-Jeghers, and Louis Bar syndromes, are also associated with the development of NMTC; however, their importance is not fully understood. 

## 3. Non-Syndromic FNMTC

### 3.1. GWAS of Thyroid Cancer

The genome-wide association study (GWAS) approach is emerging as a popular method to identify genetic factors involved in complex diseases [26]. In this review, we describe the most common germline variants associated with thyroid cancer that have been detected by GWASs. Several GWASs were conducted in patients of European descent, and some single-nucleotide polymorphisms (SNPs) are reported to be associated with thyroid cancer risk; however, only a subset of these variants have been confirmed in different populations, including Japanese, Chinese, Polish, and British, by targeted genotyping methods [27].

The first GWAS of thyroid cancer was reported in 2009. The most significant variants identified as germline candidate risk factors were SNPs located close to gene loci, including *FOXE1*, *NKX2-1*, *DIRC3*, *XRCC1*, *XRCC3*, *TITF-1*, and *TITF-2*, in patients of European descent. Signals at the *VAV3*, *INSR*, *MRSB3*, *FHIT*, *SEPT11*, and *SLC24A6* loci were only identified in Koreans [28]. These findings support the possibility of variations in the genetic factors contributing to thyroid cancer risk among different populations. Overall, the potential role of these various polymorphisms in the development of NMTC requires further characterization, and the molecular background of these polymorphisms has yet to be elucidated [26]. 

Additional studies assess five SNPs (rs965513, rs944289, rs116909374, rs2439302, and rs966423) in 1216 patients with PTC and 1416 controls, along with the expression of seven genes (*PTCSC3*, *MBIP*, *NKX2-1*, *FOXE1*, *DIRC3*, *PTCSC2*, and *NRG1*) located near to the genotyped SNPs in 73 paired PTC tumor and adjacent normal tissues. The study revealed associations between risk alleles of rs965513 and rs2439302 and more aggressive disease (larger tumor size, extrathyroidal expansions, and multifocality status). This study proved that germline variants predispose to PTC development and poor clinical outcome. In addition, expression levels of *MBIP* and *NKX2-1* (important genes in thyroid organogenesis) correlate with T stage and N1 stage, respectively [29]. Japanese researchers studied five cancer related SNPs [rs966513 (9q22.33, *FOXE1*), rs944289 (14q13.3, *PTCSC3*), rs2439302 (8p12, *NRG1*), rs1867277 (9q22.23, *FOXE1*), and rs6983267 (8q24, *POU5F1B*)] in a cohort of 959 cases with follicular adenoma (FA), 535 cases with PTC, and 2766 controls. They detected significant associations between FA and rs944289 (*p =* 0.002) and rs2439302 (*p =* 0.033). Furthermore, the following SNPs were associated with PTC: rs965513 (*p =* 4.21 × 10^−4^), rs944289 (*p =* 0.003), rs2439302 (*p* = 0.003), and rs1867277 (*p =* 1.17 × 10^−4^. These authors also noted a significant correlation between rs2439302 genotype and lower expression of *NRG1* in normal thyroid and PTC tumor tissue. This work highlighted a common risk SNP (rs944289) for benign and malignant tumors of the thyroid [30]. 

The *SMAD* family member 3 gene (*SMAD3*) shows higher expression in the thyroid than in the majority of other tissues, supporting a potential role for this factor in predisposition to thyroid cancer. Recently, Wang et al. (2018) revealed functional roles for two single-nucleotide polymorphisms (rs17293632 and rs4562997), located in separate introns of *SMAD3*, which maps to the 15q22 locus, and had previously been identified by GWAS as associated with PTC. Further, these authors investigated the downstream mechanisms by which alterations of *SMAD3* contribute to thyroid cancer susceptibility [31]. 

### 3.2. Genes and loci Associated with Non-Syndromic FNMTC

Hereditary factors contributing to the unfavorable course of FNMTC remain poorly understood; therefore, considerable efforts are being made to identify contributing loci. Understanding the molecular risk factors and prognosis of this disease would allow the evaluation of patient prognosis, which would significantly assist in managing the treatment and methods of monitoring patients. Research carried out to date has identified fourteen genes associated with a vulnerability to the development of FNMTC not related to hereditary syndromes (Table 2). In addition, seven chromosomal loci (1p13.2–1q21, 1q21, 2q21, 6q22, 8p23.1–p22, 8q24, and 19p13.2) involved in FNMTC susceptibility have been mapped, where the causal genes remain to be identified [24,32]. 

#### 3.2.1. *DICER1*

Changes in the *DICER1* gene have been identified as potentially influencing the development of NMTC. *DICER1* maps to the chromosome 14q32 locus, and the DICER1 protein is a member of the ribonuclease (RNase) III family that cleaves small non-coding RNA (miRNA) precursors to generate mature miRNAs, which regulate gene expression post-transcriptionally. Pathogenic germline *DICER1* mutations cause DICER1 syndrome, which is associated with a predisposition to various tumors and is characterized by multinodular goiter (MNG), particularly in children. 

Wasserman et al. (2018) showed that, in patients with PTC, changes in the *DICER1* gene are more frequent in diagnoses in children than in adults; however, they did not influence the aggressiveness of the disease course [34]. Further, Rutter et al. (2016) suggested that patients with the germline change c.5441C>T (p.S1814L) in *DICER1* are at an increased risk of MNG and DTC. More frequent screening may be warranted in *DICER1* families who have a first-degree relative with DTC [33]. 

#### 3.2.2. *FOXE1*, *PTCSC2*, *MYH9*

Forkhead box E1 (*FOXE1*), also known as thyroid transcription factor 2, is highly expressed in thyroid follicular cells, and is essential for thyroid gland formation and development [50]. Recently, *FOXE1* has been implicated in numerous types of cancer, including PTC [51]. Studies conducted on the Icelandic population and people of European origin indicate that the simultaneous occurrence of two polymorphic changes (rs944289 and rs965513) in the vicinity of the gene encoding *FOXE1* increases the risk of PTC and FTC development [9]. Allele [A] of rs965513 was identified as a risk factor predisposing to PTC at the 9q22 locus by GWAS. Further studies discovered a novel long intergenic noncoding RNA gene within the 9q22 locus, named papillary thyroid cancer susceptibility candidate 2 (*PTCSC2*). Allele [A] of rs965513 is correlated with low expression levels of the *PTCSC2* unspliced transcript, *FOXE1* (implicated in thyroid development), and *TSHR*, in unaffected thyroid tissue, but not in PTC tumors. By contrast, *PTCSC2* is down-regulated in PTC. Levels of the *PTCSC2* unspliced transcript are associated with age and chronic lymphocyte thyroiditis [52]. Further studies conducted on patients with PTC and controls narrowed the 9q22 locus to an approximately 33-kb linkage disequilibrium block, containing rs965513. In this block at least three regulatory elements were detected that function as enhancers, and which harbor additional SNPs (rs7864322, rs12352658, rs7847449, and rs10759944). Variants of these SNPs impact differential enhancer and/or transcription factor binding activities. These elements interact with a promotor region shared by *PTCSC2* and *FOXE1. PTCSC2* has tumor suppressor functions by inhibiting PTC cell motility and invasion through the suppression of S100A4 transcription [53]. Lately, additional studies have revealed that myosin-9 (*MYH9*) binds to the lncRNA gene, *PTCSC2*, and suppress the expression of *FOXE1*, which regulates the p53 pathway in thyroid cells. This mechanism of regulation underlies the predisposition to PTC in carriers of the rs965513 SNP [54].

#### 3.2.3. *PTCSC3*

Further work leads to the detection of the long noncoding RNA, PTC susceptibility candidate 3 (*PTCSC3*), which is located downstream of rs944289 at 14q13.3. Interestingly, *PTCSC3* expression is downregulated in PTC thyroid tumor tissue, and strongly suppressed by the T allele of rs944289. This SNP is located in a binding site for CCAAT/enhancer binding proteins (C/EBP) α and β, which activate the *PTCSC3* promotor. In silico analysis predicted that the T risk allele destroys the binding site, thereby reducing any *PTCSC3* expression. Restoration of *PTCSC3* expression in thyroid cell lines inhibits cell growth, suggesting that this protein has a tumor suppressor function [49]. 

#### 3.2.4. *SRGAP1*

He et al. (2013) assess the relationship between the *SRGAP1* gene and susceptibility to PTC. Their results indicate the importance of two missense variants (c.447A>C and c.823G>A) in the *SRGAP1* gene in increasing PTC invasiveness. These variants influence CDC42 protein activity, since SRGAP1 regulates a small G-protein that acts as a signal transduction convergence point in intracellular signaling networks, mediates multiple signaling pathways and contributes to tumorigenesis [35,55]. 

#### 3.2.5. *HABP2*

Gara et al. (2015) describe the gene variant G534E in *HABP2*, which maps to chromosome 10q25.3, the overexpression of which can be detected in tumor cells from patients with FNMTC, relative to cells from the healthy regions of the thyroid gland, or patients with sporadic NMTC [36]. 

By contrast, Alzahrani et al. (2016) studied a Middle Eastern population with FNMTC, and did not detect any germline changes in the *HABP2* gene [37]. Similarly, no association is detected between this variant and NMTC risk across all histological subtypes (PTC, FTC, and Hürthle cell) in a study reported by Sahasrabudhe et al. (2015) [56]. Kowalik et al. (2017) analyze the incidence of the c.1601G>A variant of *HABP2* in a Polish population, consisting of 326 cases with PTC and 400 controls. Their results indicate no significant difference in the frequency of the c.1601G>A variant in the Polish population at an increased risk of PTC development; the variant was more common in control individuals (4.7%; 19/400) than in patients with PTC (3.7%; 12/326). In addition, no correlations were identified between the G534E variant and clinical and pathological disease features, response to primary treatment, or clinical status [38]. 

#### 3.2.6. DNA Double-Strand Break Repair Genes (*ATM-BRCA1-CHEK2*) 

There is evidence to suggest that ionizing radiation directly influences the risk of differentiated thyroid and breast cancers. Ionizing radiation primarily promotes carcinogenesis via its ability to induce DNA double-strand breaks, which pose a challenge for cellular DNA repair pathways. The functionality of the *ATM-BRCA1-CHEK2* DNA repair pathway is affected by polymorphisms and mutations within these genes, and these underlie inefficient DNA repair, leading to tumorigenic changes within cells. In mammalian cells, DNA double-strand breaks activate the *ATM* kinase, which phosphorylates and activates *CHEK2*. Subsequently, *CHEK2* phosphorylates *BRCA1* and triggers DNA repair or, if this fails, leads to apoptosis [39]. Wójcicka et al. (2014) conducted a study to clarify the roles as factors predisposing to PTC of the following polymorphic changes: *ATM*, D1853N; *BRCA1*, E1038G; and *CHEK2*, I157T. The authors demonstrate significant associations of rs17879961 in *CHEK2* and rs16941 in *BRCA1* with susceptibility to PTC. By contrast, no such relationship is detected for rs1801516 in *ATM* [39]. Siołek et al. (2015) detect a statistically significant correlation between carrier status for the *CHEK2* gene mutations 1100delC, IVS2 + 1G>A, del5395, and I157T and increased risk of PTC. To characterize the association of *CHEK2* mutations with thyroid cancer, they genotyped 468 unselected patients with PTC and 468 (matched) cancer-free controls. A *CHEK2* mutation was detected in 73 of the 468 (15.6%) unselected patients with PTC, relative to 28 of 460 (6.0%) age- and sex-matched controls. Overall, their results suggest that *CHEK2* mutations predispose to papillary thyroid cancer and increase the risk of PTC co-occurring with breast cancer [40]. Further, Akulevich et al. (2009) and Dombernowsky et al. (2008) suggest that polymorphic changes in the *ATM* gene increase the risk of sporadic PTC and DTC in the Caucasian population [41,42]. Gu et al. (2014) analyze the relationship between polymorphic changes in *ATM* and an increased risk of PTC in the Asian population. The results indicate that, of the four polymorphisms studied (rs664677, rs373759, rs4988099, and rs189037), only rs373759 was associated with an increased risk of PTC development [43].

#### 3.2.7. *RASAL1*

The protein encoded by *RASAL1* is member of the GAP1 family of GTPase-activating proteins. Acting as a suppressor of oncogenic RAS function, *RASAL1* protein enhances the weak, intrinsic GTPase activity of RAS proteins, resulting in an inactive GDP-bound form of RAS, thereby allowing control of cellular proliferation and differentiation [57]. Xing et al. (2013) study how alternative RAS signaling-related genes affect thyroid tumorigenesis. Compared with normal human thyroid tissue, the RAS GTPase-activating protein gene, *RASAL1*, is commonly, but differentially, somatically mutated or hypermethylated in thyroid cancers. Sequence analysis of thyroid cancer samples for somatic mutations in *RASAL1* predominantly identify mutations in FTC tumors [58]. Given these recent findings, Ngeow et al. (2014) explore the prevalence of germline *RASAL1* mutations in a subset of patients with Cowden syndrome who have thyroid cancer. They genotyped 155 patients for germline *RASAL1* mutations, among which 39 had *PTEN* mutations and 116 did not. Of the 116 patients, 53 (46%) had either FTC or follicular-variant PTC, 54 (47%) had PTC, 7 (6%) had Hürthle cell thyroid cancer, and 1 had ATC, while among the 39 patients, one-third (13/39) had FTC or follicular-variant PTC. 

Among all 155 patients, *RASAL1* germline alterations suspected to be deleterious were detected in two patients. Interestingly, both were patients without *PTEN* mutations who had FTC and had the same pathogenic missense variant in *RASAL1*, R328W, in exon 11. Ngeow et al. (2014) did not detect any deleterious germline *RASAL1* alterations in patients with thyroid cancer with simultaneous mutations in *PTEN*. Based on these findings, germline mutations in the *RASAL1* gene may be a risk factor for the development of FTC in PTEN mutation-negative patients and those with Cowden syndrome [44].

#### 3.2.8. *SRRM2*

Tomsic et al. (2015) use a combination of genetic methods and detect mutation in a single candidate gene, SRRM2. SRRM2 is a splicing factor, and promotes exon enhancer-dependent splicing by forming multiple critical interactions with factors bound directly to pre-mRNA. Analysis leading to identify a variant c.1037C > T (S346F) in the splicing gene SRRM2, was making it a leading candidate mutation contributing to the development of PTC. Authors speculate that the mutated protein affects the splicing of at least one of the genes specifically expressed in the thyroid or involved in a thyroid-specific pathway, thereby leading to PTC formation [45]. 

#### 3.2.9. *XRCC1*

*XRCC1* encodes a protein with an important role in the base excision repair (BER) pathway for single-strand DNA break repair and maintenance of genetic stability. Ryu et al. (2011) perform a genetic analysis of two known polymorphisms of the *XRCC1* gene, R194W and R399Q, in a Korean sample. The frequencies of the *XRCC1* R194 genotype are 38.7% in the cancer group and 49.0% in the control group, while those of *XRCC1* R399 genotype are 15.3% and 19.0%, respectively. There are no sex- or age-associated statistically significant differences with regard to the risk of PTC for the two *XRCC1* polymorphisms. The molecular consequences of the two non-synonymous polymorphisms in *XRCC1* analyzed by Ryu et al. (2011) are not yet fully understood [46].

#### 3.2.10. *TITF-1/NKX2.1*

*TITF-1/NKX2.1* comprises two exons that encode a 42-kDa thyroid transcription factor-1, TTF-1, which activates the transcription of thyroglobulin, thyroperoxidase, and the thyrotropin receptor [24]. Ngan et al. (2009) examine DNA extracted from blood samples from 20 patients with MNG/PTC, 284 with PTC, and 349 control subjects, for mutations in *TITF-1/NKX2.1.* Targeted DNA sequencing reveals a germline *TITF-1/NKX2.1* A339V mutation in four patients with PTC and a history of MNG. Of these four patients, two have a positive family history of PTC, and the pattern of inheritance was autosomal dominant in both families. In the first family, three cases carry this germline mutation, each of which was affected with PTC and MNG, MNG, or PTC with MNG. In the second family, two members have this germline mutation, both of whom present with PTC and MNG or MNG [47]. Cantara et al. (2010) analyze 63 PTC patients for the presence of the A339V mutation in *TITF-1/NKX2.1*. They suggest that the mutation correlates with a predisposition to the development of a familial form of thyroid cancer; however, their results show that all of the 63 patients with PTC tested have the wild-type allele at this locus, providing no support for a role for the A339V mutation in FNMTC development [48]; hence, the utility of the A339V TTF-1 mutation as a susceptibility gene for the development of PTC remains uncertain.

## 4. Molecular Analysis of Germline Changes as Potential Prognostic Factors in PTC

Over the past five decades, the mortality rate for patients with PTC has significantly decreased. Moreover, patients with PTC have a very good prognosis, since approximately 95% survive for 10 years. The vast majority of patients with PTC are diagnosed with benign tumors, while an analysis of autopsy results, focused on papillary thyroid microcarcinoma, finds that this condition could be detected in 30% of people who die of other causes. 

Hence, the main clinical challenge regarding PTC is to avoid overdiagnosis and any overtreatment of people with low-grade disease or benign thyroid nodules. By contrast, rapid identification of aggressive cases is vital, as the cause of death is often metastasis [59,60]. The poor understanding of predictive germline factors is a very serious problem, and knowledge of these would allow an estimation of patient prognosis before treatment. Unlike lesions, characteristic tumor gene polymorphisms can be determined from patient peripheral blood samples long before surgery. Świerniak et al. (2016) analyze the relationship between the genetic polymorphism rs966423 in *DIRC3* and the mortality in patients diagnosed with DTC. Their results indicate a relationship between an increased risk of death during the course of DTC and the prognostic factors, such as the rs966423 variant in *DIRC3*, male sex, age > 45 years, tumor size > 30 mm, angioinvasion, lymph node metastasis (pN1b), distant metastasis (M1), and stage IV disease [61]. Wei et al. (2015) analyze the relationship between specific polymorphisms (rs944289, rs965513, rs966423, and rs2439302) and clinical and pathological factors in patients with PTC. They also find that rs966423 in *DIRC3* is associated with tumor invasion and multifocality, and is a potential prognostic factor [62].

## 5. Significance of microRNA Polymorphisms in NMTC

Small noncoding RNA molecules (microRNAs) participate in a variety of biological processes and function as suppressors to regulate mRNA stability. SNPs within microRNA sequences may change their properties, and such SNPs are involved in the tumorigenesis of diverse malignancies, with aberrant levels of miRNAs observed in PTC cases compared with healthy controls. Further, microRNAs can change the formation and function of the thyroid tissues by regulating gene expression [63].

### 5.1. rs2910164

One of the most common miRNA SNPs to be reported in connection with PTC risk is rs2910164 (miR-146a) [63]. In an association study of 608 patients with PTC and 901 controls, Jazdzewski et al. (2008) show that the GC heterozygous state at rs2910164 is associated with an increased risk of acquiring PTC (odds ratio = 1.62, *p =* 0.000007), whereas both homozygous states (GG and CC) are protective, with odds ratios of 0.42 for the CC genotype (*p =* 0.003) and 0.69 for the GG genotype (*p =* 0.0006) [64]. Dong et al. (2015) investigate the association between the miRNA variants, rs2910164 (miR-146a), rs4919510 (miR-608), rs79402775 (miR-933), and rs2292832 (miR-149), and the PTC risk in a Han Chinese population. No significant association is detected between the miR-146a polymorphism and the PTC risk under the four established genetic models. Similarly, neither miR-608 nor miR-933 variants confer PTC risk under these genetic models; however, the miR-146a polymorphism is significantly correlated with an elevated risk of PTC under the heterozygous, homozygous, dominant and allelic models, based on comparisons of genotype distribution between PTC cases and healthy controls. These authors also conduct a meta-analysis, to summarize the overall effect of the miR-146a SNP on PTC risk. Nevertheless, Dong et al. (2015) report no significant association between miR-146a polymorphisms and PTC risk under any genetic model [63]. 

### 5.2. rs11077

Exportin 5 (XPO5) is an essential specific nuclear transport factor, involved in the completion of pre-miRNA transport into the cytoplasm from the nucleus. Overexpression of XPO5 is thought to lead to a higher activity of miRNAs, while a loss of XPO5 expression inhibits the nuclear export of pre-miRNAs. Wen et al. (2017) compares XPO5 expression levels in thyroid cancer and normal tissues, and then evaluates and quantifies the association between XPO5 miR-SNPs and any susceptibility to thyroid cancer in a Chinese population. Expression of XPO5 was significantly lower in thyroid cancer than in normal tissues. Further, the XPO5 rs11077 TG/GG genotype is associated with higher susceptibility to thyroid cancer. Moreover, the TG/GG genotypes correspond to lower expression of XPO5 mRNA. 

These results suggest that the XPO5 miRNA-SNP, rs11077, is a potential biomarker for thyroid cancer prediction, via its functional impact upon XPO5 expression [65]; however, there is no report of a functional connection between XPO5 and thyroid cancer incidence.

## 6. Conclusions

We note significant developments and progress in research to identify prognostic molecular factors in PTC, the knowledge of which would significantly facilitate the estimation of disease course; however, the results obtained to date do not allow any clear determination of the impact of the changes in a particular gene as a key factor in the development and prognosis of PTC. Further research is warranted to fully characterize the pathogenesis of PTC and the genetic contribution to this disease.

## Figures and Tables

**Table 1:** Syndromic familial non-medullary thyroid cancer (NMTC). (Data from Yang et al. 2016, Vriens et al. 2009, and Griffith et al. 2016).

Syndrome	Germline Genetic Alteration	Type of Thyroid Cancer	Frequency of Thyroid Lesions
Familial adenomatous polyposis (FAP)	APC mutation	PTC	1%–12% (usually women)
Cowden’s disease	PTEN mutation SDHB-D mutation, PIK3CA mutation AKT1 mutation KLLN promoter methylation SEC23B mutation	PTC (classical and follicular variant), FTC	10% Unknown
Werner syndrome	WRN mutation	PTC, FTC, ATC	18% of Japanese patients develop thyroid carcinoma
Carney complex	PRKAR1α mutation	PTC, FTC	3%
Papillary renal neoplasia	Unknown	PTC	Unknown

ATC, anaplastic thyroid cancer; FTC, follicular thyroid cancer; PTC, papillary thyroid cancer.

**Table 2:** Genes involved in the development of non-syndromic familial non-medullary thyroid cancers and the significance of the mutations in these genes as risk factors.

Gene	Type of Thyroid Cancer	Risk Factor	Reference
*DICER1*	PTC	+	Rutter et al. (2016) [33] Wasserman et al. (2018) [34]
*FOXE1,PTCSC2,MYH9*	PTC and FTC	+	Pereira et al. (2015) [9]
*SRGAP1*	PTC	+	He et al. (2013) [35]
*HABP2*	PTC	+/−	Gara et al. (2015) [36] Alzahrani et al. (2016) [37] Kowalik et al. (2017) [38]
*BRCA1*	PTC	+	Wójcicka et al. (2014) [39]
*CHEK2*	PTC	+	Wójcicka et al. (2014) [39] Siołek et al. (2015) [40]
*ATM*	PTC and DTC	+	Dombernowsky et al. (2008) [41] Akulevich et al. (2009) [42] Gu et al. (2014) [43]
*RASAL1*	FTC	+	Ngeow et al. (2014) [44]
*SRRM2*	PTC	+	Tomsic et al. (2015) [45]
*XRCC1*	PTC	+/−	Ryu et al. (2011) et al. [46]
*TITF-1/NKX2.1*	PTC	+/−	Ngan et al. (2009) [47] Cantara et al. (2010) [48]
*PTCSC3*	PTC	+/−	Jendrzejewski et al. (2012) [49]

PTC, papillary thyroid cancer; FTC, follicular thyroid cancer; DTC, differentiated thyroid cancer.
